# Supplementary material for: Microrollers flow uphill as granular media
Source: Nat Commun. 2023 Sep 20;14:5829. doi: 10.1038/s41467-023-41327-1 (PMC10511535; doi:10.1038/s41467-023-41327-1)
Supplement: Supplementary file 1 — Supplementary Information [file 41467_2023_41327_MOESM1_ESM.pdf]

Supplementary Information

Microrollers flow uphill as granular media

Samuel R. Wilson-Whitford[1], Jinghui Gao[1], Maria Chiara Roffin[1,2], William E. Buckley[1] and James F. Gilchrist[1,]*

[1]*Department of Chemical and Biomolecular Engineering, Lehigh University, Bethlehem, Pennsylvania, USA, 18015*

[2]*Department of Physics, School of Science and Technology, Nottingham Trent University, Nottingham, UK, NG11 8NS*

gilchrist@lehigh.edu

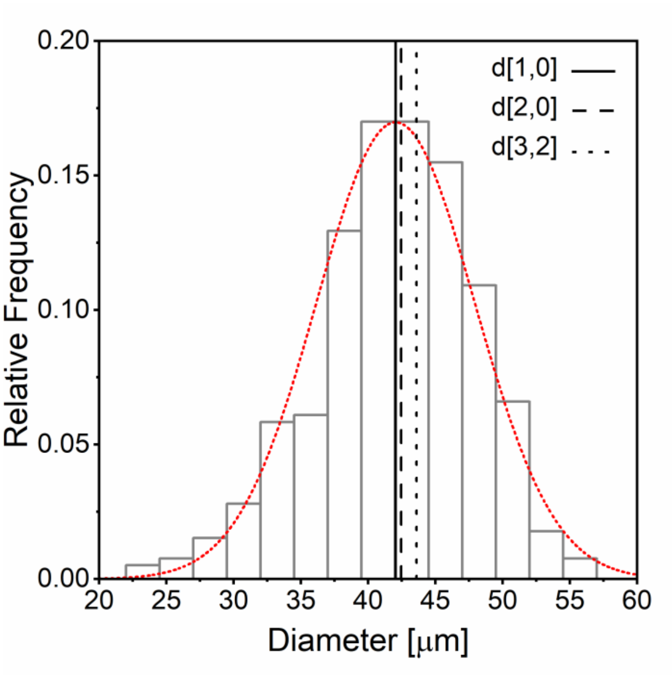

Supplementary fig. 1 Particle size distribution measured by optical microscopy over 600 particles. Solid, dashed and dotted lines show the mean particle diameter according to different averaging techniques, d[1,0] length mean, d[2,0] area mean and d[3,2] Sauter mean.

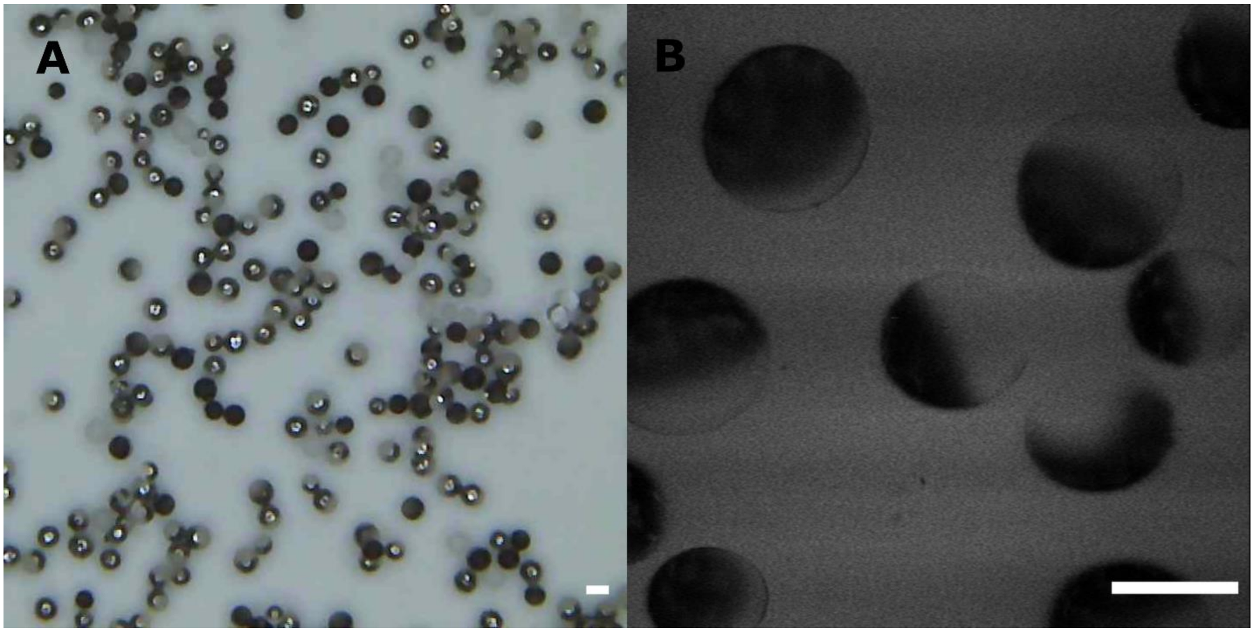

Supplementary fig. 2 Microscopy of PMMA ferromagnetic Janus particle (a) Digital microscope image (b) confocal laser scanning microscopy. Scale bars = 43.6 μm (d[3,2]).

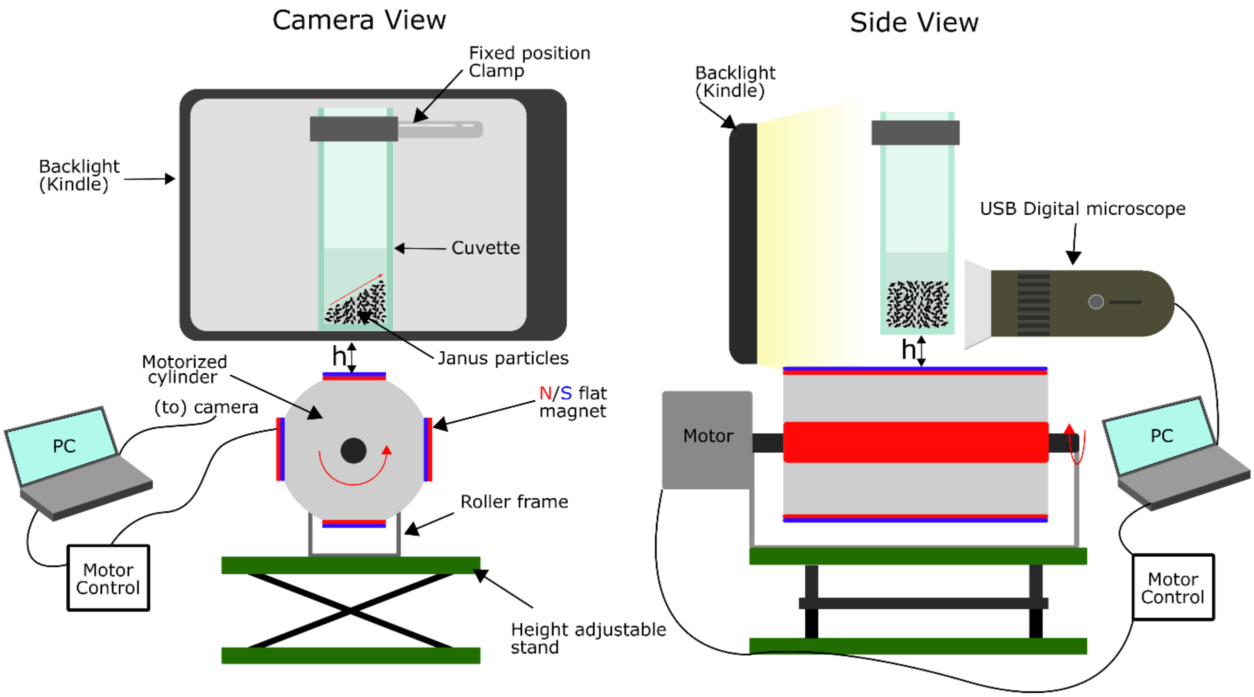

Supplementary fig. 3 Illustration of experimental set up viewed from the camera and from the side.

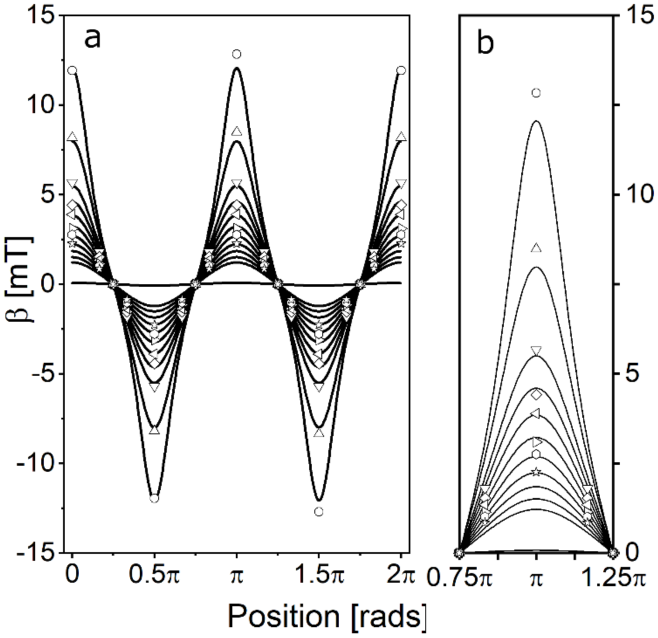

Supplementary fig. 4 Fit of experimental field strength from 0-2π. Experimental data covers distances from the magnetic surface of h = 15, 20, 25, 27.5, 30, 32.5, 35 and 37.5 mm.

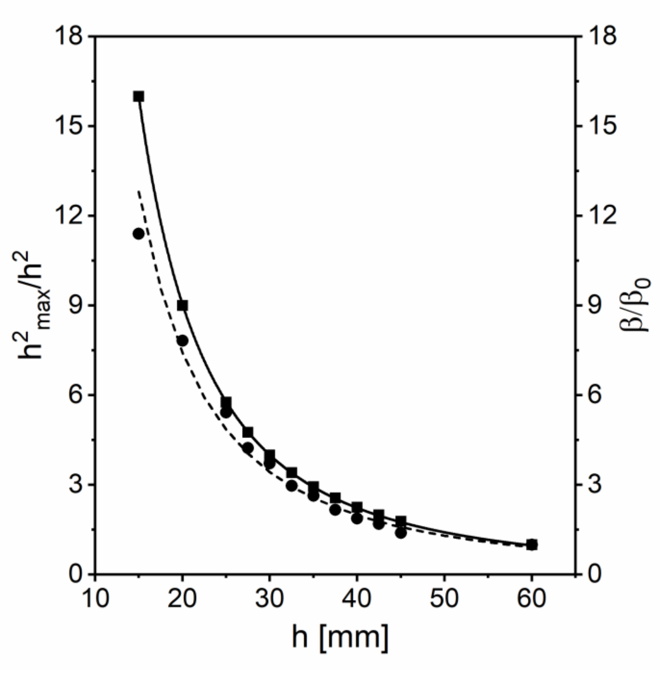

Supplementary fig. 5 Comparison of scaling of h^2_{max}/h^2 to h (square, solid line) and β/β_0 to h (circle, dash line) to show proportionality between field strength and separation from the magnetic surface. Fit $h^2 = x^{-2}$; fit $\beta = x^{-1.9}$. Here h^2_{max}/h^2 is a representation of the field strength with respect to the maximum measured magnetic field in this experiment, using h which itself is inversely proportional to the field strength, β. From this the relationship $F \propto \beta^2 \propto 1/h^4$.

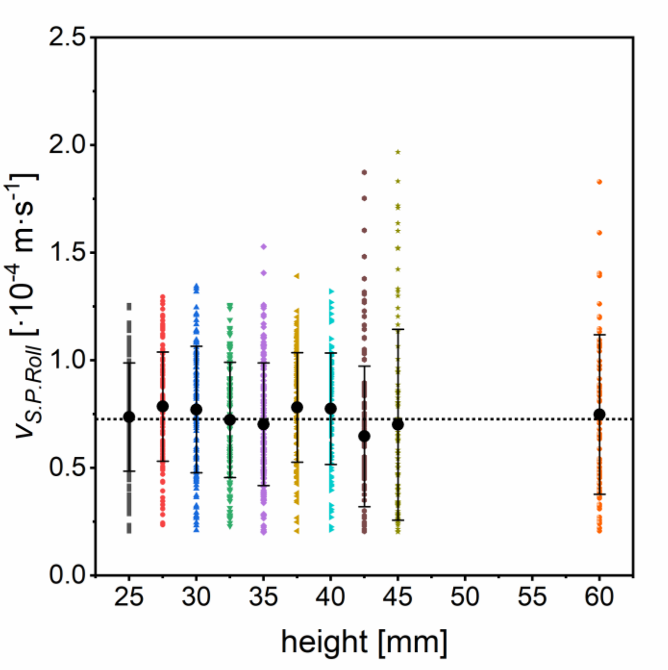

Supplementary fig. 6 Single particle rolling velocity ($v_{S.P.Roll}$) of Janus particles in dilute conditions at different separation (height, h) from the magnetic surface, calculated from multiple particle tracking algorithms. Plot shows that the rolling velocity of a single Janus particle rolling on a flat substrate, under the influence of the rotating magnetic field, is independent of the field strength, i.e. the separation from the magnet. Large black circles indicated the average and standard deviation at each height. Overall average $v_{S.P.Roll} = 7.37 \cdot 10^{-5} \pm 4.23 \cdot 10^{-6}$ m·s^{-1}. Wide standard deviations are related to size distribution of the Janus particles. $\rho_{ethanol} = 789$ kg·m^{-3}; $\mu_{ethanol} = 0.001095$ Ns·m^{-2};

$$Re = \frac{\rho u R}{\mu} \approx 1.04 \cdot 10^{-3}$$

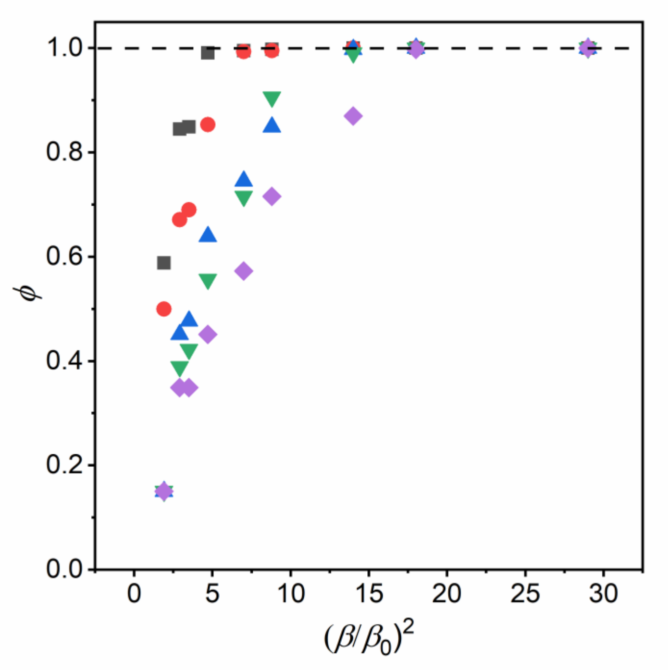

Supplementary fig. 7 Degree of fluidization, ϕ, in the bed with increased magnetic field strength, $(\beta/\beta_0)^2$. For samples $\Delta/2a$, 9.5 (square, black) 18.5 (circle, red) 26.0 (upward triangle, blue) 31.0 (sideward triangle, green) 39.0 (diamond, purple).
