# Supplementary material for: Microrollers flow uphill as granular media
Source: Nat Commun. 2023 Sep 20;14:5829. doi: 10.1038/s41467-023-41327-1 (PMC10511535; doi:10.1038/s41467-023-41327-1)
Supplement: Supplementary file 3 — Description of Additional Supplementary Files [file 41467_2023_41327_MOESM3_ESM.pdf]

<u>Description of additional supplementary files</u>

Supplementary Movie 1 : Uphill Granular Flow V1 Uphill heaping of granular scale Janus particles under the influence of a rotating magnetic field, $(\beta/\beta_0)^2 = 1.9$, when $\chi \ll 1$ and bed depth, $\Delta/2a = 26.0$.

Supplementary Movie 2 : Uphill Granular Flow V2 Uphill heaping of granular scale Janus particles under the influence of a rotating magnetic field, $(\beta/\beta_0)^2 = 7$, when $\chi < 1$ and bed depth, $\Delta/2a = 26.0$

Supplementary Movie 3 : Uphill Granular Flow V3 Uphill heaping of granular scale Janus particles under the influence of a rotating magnetic field, $(\beta/\beta_0)^2 = 18$, when $\chi = 1$ and bed depth, $\Delta/2a = 26.0$

Supplementary Movie 4 : Initiation, from rest, of uphill heaping of granular scale Janus particles under the influence of a rotating magnetic field, $(\beta/\beta_0)^2 = 3.5$ and bed depth, $\Delta/2a = 31.0$. Duration represents 3 minutes of heaping.
