# Supplementary figures and images for: Microrollers flow uphill as granular media
Source: Nat Commun. 2023 Sep 20;14:5829. doi: 10.1038/s41467-023-41327-1 (PMC10511535; doi:10.1038/s41467-023-41327-1)

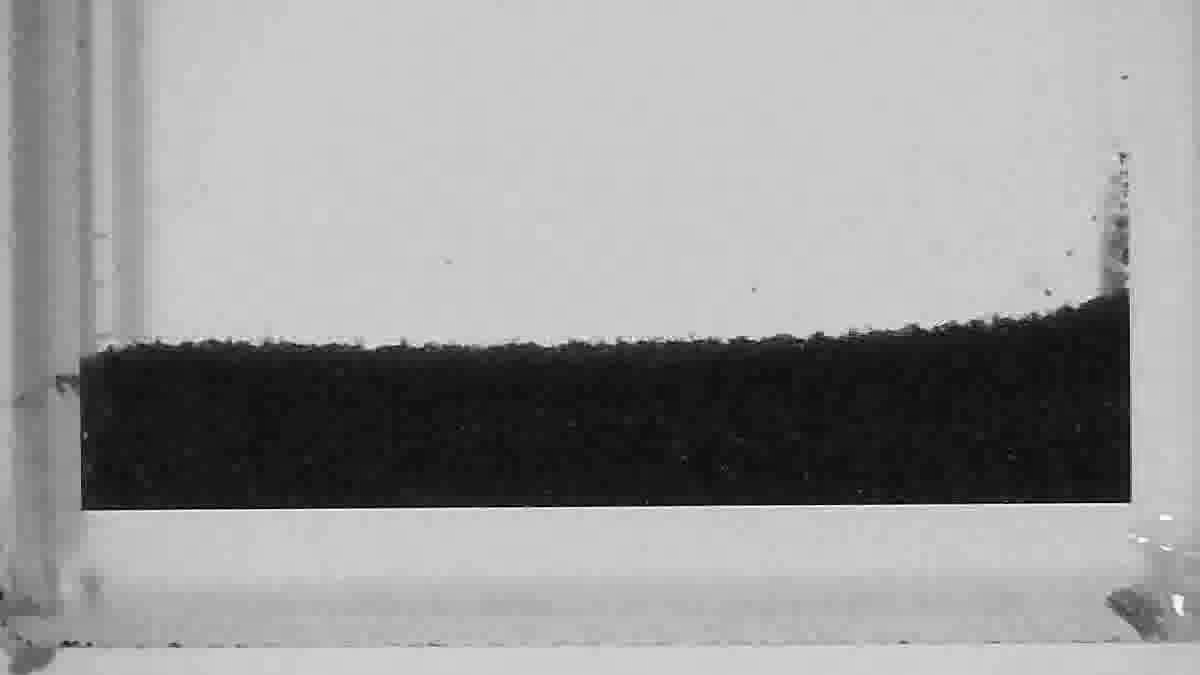

Supplement: Supplementary file 7 — Supplementary Movie 4 [file 41467_2023_41327_MOESM7_ESM.gif]
